# Editorial

**DOI:** 10.1120/jacmp.v3i3.2559

**Published:** 2002-06-01

Once again it is time to announce the awards for the best papers published in the JACMP during the previous year. These papers were selected by the Awards and Honors Committee of the American College of Medical Physics, and the awards were presented at the annual meeting of the College held June 1‐6.

The LAP Award for Excellence for the best radiation oncology physics paper went to Maria F. Chan, Albert YC Fung, Yu‐Chi Hu, Chen‐Shou Chui, Howard Amols, Marco Zaider, and David Abramson for “The measurement of the three dimensional dose distribution of a ruthenium‐106 opthalmological applicator using magnetic resonance imaging of BANG polymer gels,” JACMP, Vol. 2, No. 2, Spring 2001.

The RIT Award for Excellence for the best diagnostic imaging paper went to Eugene Mah, Ehsan Samei, and Donald J. Peck for “Evaluation of a quality control phantom for digital chest radiography,” JACMP, Vol. 2, No. 2, Spring 2001.

The Elekta Award for Excellence for the best professional medical physics paper went to William Que for “Radiation safety issues regarding the cremation of the body of an I‐125 prostate implant patient,” JACMP, Vol. 2, No. 3, Summer 2001.

Congratulations to all these authors for their fine work and papers. Each paper receives a $500 check and each author receives a framed certificate. The Journal continues to be extremely grateful to LAP of America, Radiological Imaging Technology, and Elekta for making these awards possible.

The CD‐ROM of Volumes 1 and 2 of the JACMP has now been produced. The CD was distributed to all members of the American Association of Physicists in Medicine in the June mailout. Our thanks again to Radiological Imaging Technology for sponsoring the production of this disc.

Peter R. Almond, Ph.D.

Editor‐in‐Chief

